# Supplementary material for: Hybrid Models and Biological Model Reduction with PyDSTool
Source: PLoS Comput Biol. 2012 Aug 9;8(8):e1002628. doi: 10.1371/journal.pcbi.1002628 (PMC3415397; doi:10.1371/journal.pcbi.1002628)
Supplement: Text S4 — Complete source code for the PyDSTool package (version 0.88.120504). Includes API documentation and help files linking to web pages. This file is identical to the current public release on Sourceforge.net. (ZIP) [file pcbi.1002628.s004.zip › PyDSTool/html/PyDSTool.MProject.extModelInterface-class.html]

xml version="1.0" encoding="ascii"?


PyDSTool.MProject.extModelInterface


| Home | Trees | Indices | Help | PyDSTool |
| --- | --- | --- | --- | --- |

Package PyDSTool ::
Module MProject ::
Class extModelInterface

[hide private]

[frames] | no frames]

# Class extModelInterface

source code

```
 object --+        
          |        
dsInterface --+    
              |    
 ModelInterface --+
                  |
                 extModelInterface
```

---

Interface from a trajectory of numerical data and test conditions
providing external evaluation criteria for a model. Optional conditions
(object) argument used to specify these criteria.


|  |  |
| --- | --- |
| Instance Methods | [hide private] |

|  |  |
| --- | --- |
| \_\_init\_\_(self, traj=None, conditions=None, compatibleInterfaces=None)  x.\_\_init\_\_(...) initializes x; see x.\_\_class\_\_.\_\_doc\_\_ for signature | source code |

|  |  |
| --- | --- |
| set\_test\_traj(self, traj)  Do any user-defined preprocessing to the given trajectory, including converting it to a different type of trajectory. | source code |

|  |  |
| --- | --- |
| ensure\_has\_test\_traj(self)  Never needs to recompute trajectory as it is fixed, so always returns False. | source code |

|  |  |
| --- | --- |
| has\_test\_traj(self) | source code |

|  |  |
| --- | --- |
| get\_test\_traj(self, force=False)  Called by another interface. | source code |

**Inherited from `ModelInterface`**:
`AuxVars`,
`Jacobian`,
`JacobianP`,
`MassMatrix`,
`Rhs`,
`__call__`,
`evaluate`,
`get`,
`postprocess_test_traj`,
`prepare_conditions`,
`set`,
`setup_conditions`

**Inherited from `ModelInterface`** (private):
`_get_initiator_cache`

**Inherited from `dsInterface`**:
`query`

**Inherited from `object`**:
`__delattr__`,
`__getattribute__`,
`__hash__`,
`__new__`,
`__reduce__`,
`__reduce_ex__`,
`__repr__`,
`__setattr__`,
`__str__`


|  |  |
| --- | --- |
| Class Variables | [hide private] |

**Inherited from `ModelInterface`** (private):
`_trajname`

**Inherited from `dsInterface`** (private):
`_querykeys`,
`_setkeys`


|  |  |
| --- | --- |
| Properties | [hide private] |

**Inherited from `object`**:
`__class__`


|  |  |
| --- | --- |
| Method Details | [hide private] |

|  |  |
| --- | --- |
| \_\_init\_\_(self, traj=None, conditions=None, compatibleInterfaces=None)  *(Constructor)* | source code |

x.\_\_init\_\_(...) initializes x; see x.\_\_class\_\_.\_\_doc\_\_ for
signature

Overrides: object.\_\_init\_\_: *(inherited documentation)*

|  |  |
| --- | --- |
| get\_test\_traj(self, force=False) | source code |

```
Called by another interface.
Optional force argument is ignored for this class, as the
 trajectory is fixed.
```

Overrides: dsInterface.get\_test\_traj

  


| Home | Trees | Indices | Help | PyDSTool |
| --- | --- | --- | --- | --- |

|  |  |
| --- | --- |
| Generated by Epydoc 3.0.1 on Fri May 4 15:24:06 2012 | http://epydoc.sourceforge.net |
